# Analysis and prediction of schizophrenia patients based on high-order graph attention generative adversarial networks

**DOI:** 10.1038/s41598-025-15602-8

**Published:** 2026-02-03

**Authors:** Guimei Yin, Mengzhen Yin, Guangxing Guo, Jie Yuan, Xiaoxiao Ma, Lin Wang, Peng Zhao, Dongli Shi, Yanli Zhao, Zilong Zhao, Bin Wang, Shuping Tan

**Affiliations:** 1 College of Computer Science and Technology, Taiyuan Normal University, Jinzhong, 030619 China; 2 Institute of Big Data Technology Analysis and Application, Taiyuan Normal University, Jinzhong, 030619 China; 3 Department of Radiology, Shanxi Provincial People’s Hospital, Taiyuan, 030012 China; 4 Psychiatry Research Center, Beijing Huilongguan Hospital, Peking University Huilongguan Clinical Medical School, Beijing, 100096 China; 5 School of Chemical Engineering and Technology, Sun Yat-sen University, Zhuhai, 519080 China; 6 College of Computer Science and Technology, Taiyuan University of Technology, Jinzhong, 030600 China

**Keywords:** Graph attention networks, Generating adversarial networks, Image quality coefficient, Persistence image, Schizophrenia, Computational neuroscience, Computational biology and bioinformatics, Neurology

## Abstract

Generative Adversarial Networks, a popular deep learning method, have achieved excellent performance in both classification and prediction tasks. However, there have been relatively few applications of generative adversarial networks to EEG data. To study the effect of high-order brain functional networks on schizophrenia patients, a high-order graph attention generative adversarial network prediction model is proposed, and the generator of the model utilizes graph attention networks and long short-term memory networks to capture the high-order topological features of persistence images for early diagnosis and prediction of schizophrenia patients. The research results on the five frequency bands of schizophrenia show that the proposed prediction model performs best in the Theta frequency band, with AUC and MAP values reaching 93.5% and 93.0%, respectively, and an average accuracy of 91.5%, both of which are superior to the selected comparison methods. Moreover, the image quality coefficient is used to quantify the realism and clarity of the images generated by the model. the image quality coefficients of schizophrenia patients were significantly correlated with the PANSS total scores in the Gamma and Theta bands, which provided a new idea for generative adversarial networks in the prediction of schizophrenia high-order topological features.

**Supplementary Information:**

The online version contains supplementary material available at 10.1038/s41598-025-15602-8.

## Introduction

Schizophrenia is a severe mental disorder characterized by high relapse rates and the necessity for long-term pharmacological treatment. Approximately 24 million people are affected globally^1^ and the number of cases in China is projected to reach 8.604 million by 2025, representing a growth rate of 1.3%, which has become a key challenge for clinical treatment^2,3^.

In recent years, machine learning and deep learning techniques have significantly advanced in the analysis of EEG data. De Miras et al.^4^ evaluated five machine learning methods-k-nearest neighbors, logistic regression, decision trees, random forests, and support vector machines-for the diagnosis of schizophrenia, identifying specific brain regions associated with the disorder and providing a biological basis for this association. Shalbaf et al.^5^ used an automated method of deep convolutional neural networks based on transfer learning for diagnosing schizophrenia patients from healthy controls, a pre-trained deep convolutional neural network, which effectively extracted complex features with minimal human intervention. These methods not only improve diagnostic accuracy but also offer potential avenues for individualized treatment. Although machine learning and deep learning approaches show great potential in EEG research, their respective strengths and limitations, such as small sample sizes and feature selection challenges, require further investigation. Generative Adversarial Networks (GANs) have been widely used in image enhancement, image synthesis, and medical applications^6–8^. In image enhancement, the generator produces an image that is perceptually indistinguishable from the original image by improving the contrast, color saturation, sharpness, detail restoration, and noise removal of the image, which enhances the visual effect and quality of the image; In image synthesis, the generator produces indistinguishable synthetic images and the discriminator identifies the authenticity of the image by comparison; In the medical field, GAN removes noise and artifacts to enhance the quality of medical images, thus assisting doctors in analyzing medical images for pathological judgments and diagnostic comparisons. Som et al.^9^ directly generated persistence images from input data and designed two convolutional neural network architectures for processing multivariate time series signals and multi-channel images. The results indicate that persistence images can be integrated into supervised deep learning architectures while significantly accelerate feature extraction. Chepushtanova et al.^10^ vectorized persistence diagrams as persistence images, and various machine learning techniques applied to persistence images have achieved high accuracy in classification tasks on multiple datasets. These techniques perform better on persistence images than on persistence diagrams.

GAN methods have proven to be successful in prediction tasks, where such methods learn the characteristics of the time series and thus predict outcomes. For example, Yu et al.^11^ proposed a conditional generative adversarial network with a long short-term memory (LSTM) structure to capture both the spatial and temporal variations of cab hotspots and thus achieve prediction. Lei et al.^12^ proposed a nonlinear model combining graph convolutional networks and GANs, where the generator contains graph convolutional networks and LSTMs capturing both topological and temporal features of weighted dynamic networks for prediction. Vuletić et al.^13^ studied the application of GAN in probabilistic forecasting of financial time series. However, the current approach suffers from several problems: (1) the results of the model are prone to the loss of important topology due to the lack of high-order graph information in the node representations learned by the GCN; (2) the generated images by the GAN fails to take into account the importance of the different nodes between them in the graph structure.

Graph attention networks (GAT) have been recognized as an important analytical method for graph structure analysis^14,15^. In recent years, there have been some scholars constantly combining graph attention networks with deep learning. Zhao et al.^16^ proposed a dynamic graph convolutional network dynamically computing graph features and utilizing sparse brain region connections for training. Xu et al.^17^ proposed a graph context attention network that employed high-order attention mechanisms and adversarial regularization constraints. This network performed representation learning by aggregating both low-order and high-order information. He et al.^18^ proposed a high-order graph attention network that adaptively aggregated node features from multi-hop neighbors through an attention mechanism. Guan et al.^19^ proposed a high-order graph attention network vector cellular automaton model. This model constructed a graph structure based on the topological adjacency relationships between land parcels and expanded the first-order adjacency relationships to high-order ones to capture spatial features. The emergence of high-order graph attention networks solves the drawbacks of graph structure. In addition, persistence images are a method for translating persistent homotopy results from topological data analysis into a structured numerical representation. They form color images by mapping the topological features in the persistence image to a two-dimensional pixel space, where the pixel intensities reflect the persistence intensities of the corresponding topological features. There representations not only preserves the topological properties of the original data, but also provides regularized inputs suitable for processing by deep learning models. Based on the complementary advantages of these two techniques, this paper proposes the Persistence Image Graph Attention Generative Adversarial Network (PIGAT-GAN) model. First, the EEG time series are extracted from the higher-order Persistence Image (PI) by topological data analysis methods^20,21^ and the PI is used as an input to the generator. Then, generators containing GAT and LSTM are designed to capture the evolutionary features of high-order topological features and predict the high-order PI at the next moment. Finally, the discriminator discerns the difference between the predicted PI and the true PI and feeds back to the generator to continuously improve the efficiency of PI prediction. The contributions of this paper are as follows:

1. The application of high-order PIs in schizophrenia research is explored, revealing the importance of high-order topological features.
2. The problem of insufficient data sample size is effectively solved by using GAN.
3. The PIGAT-GAN model is proposed, which significantly enhances the characterization of high-order topological features and improves the prediction accuracy of schizophrenia.

## Methods

**Fig. 1:**
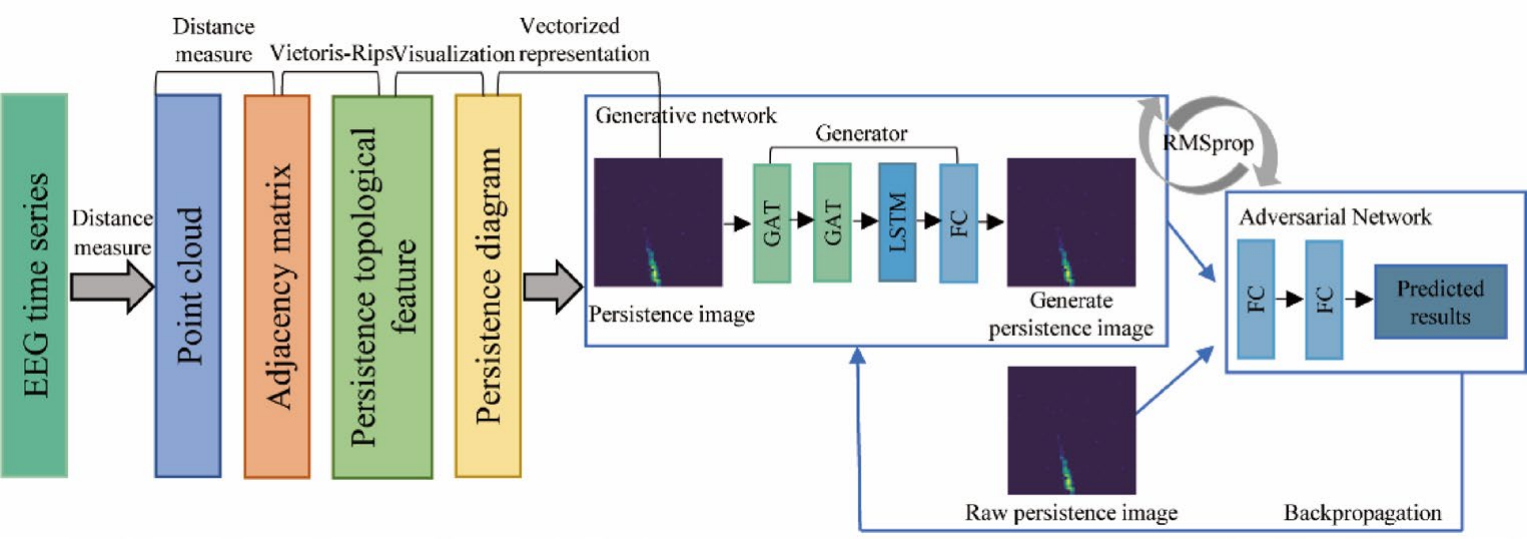
Structure of the PIGAT-GAN model.

The PIGAT-GAN modeling system contains three modules: higher-order PI, generator, and discriminator. As shown in Fig. 1. Specifically, first the EEG time series are transformed into a persistence diagram employing a topological data analysis method and further vectorized to be represented as a high-order PI, which is then used as an input to the generator, and an exhaustive description of this transformation step is given in Sect. “High-order persistence images”; Immediately after that, the GAT in the generator controls the edges and nodes of the high-order PIs, and the LSTM captures the temporal dependencies in the PI and generates a sequence of images with temporal continuity, from which the generator predicts the high-order PI at the next moment. Finally, the discriminator evaluates the difference between the predicted high-order PI and the actual high-order PI by comparing the predicted high-order PI with the actual high-order PI and feeds it back to the generator, thus iterating in a loop to continuously optimize the prediction accuracy and realism of the model.

### High-order persistence images

The experiment uses 160 s of EEG signals from each subject for frequency division into five different bands. Current studies have show that most EEG researchers use either 2 or 4-segment lengths in spectral analysis, and there is no significant difference between these two choices^22^ however, the use of a 4-s segment length allows for more efficient extraction of features of the EEG signal^23,24^ and therefore we divide the EEG signals of each subject, under each frequency band, into consecutive non-overlapping time windows with a segment length of 4 s.

**Fig. 2:**
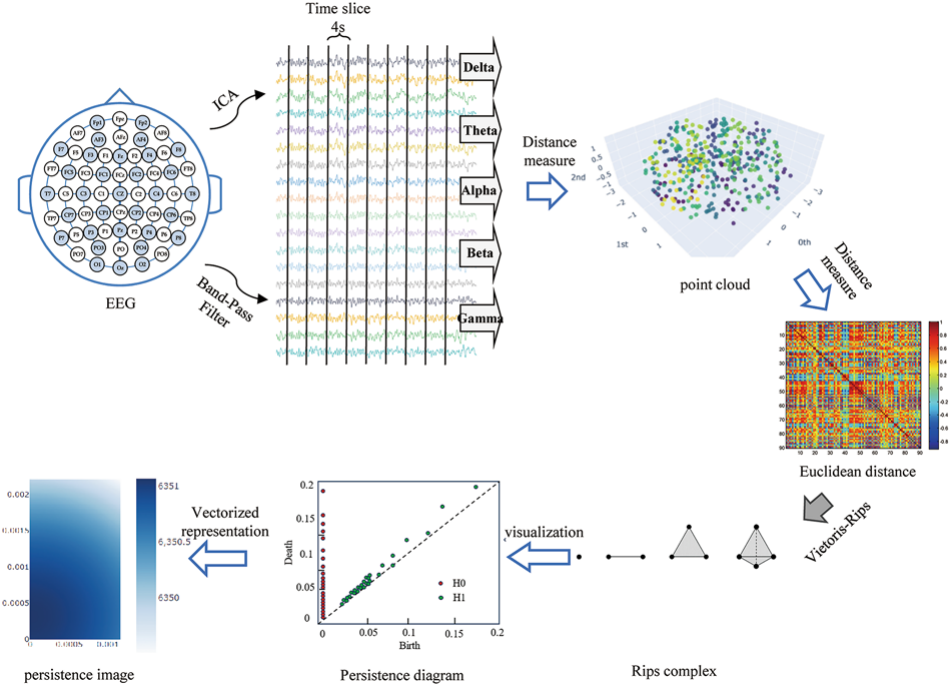
High-order persistence image structure.

The EEG time series are first subjected to average reference processing, filtering, independent component analysis, and interpolation^25^ and the processed EEG data is divided into five frequency bands according to a segment length of 4 s, Delta (1–3 Hz), Theta (4–7 Hz), Alpha (8–12 Hz), Beta (13–30 Hz), and Gamma (31–49 Hz) five frequency bands, and for each band separately, the data is converted into a point cloud in a high-dimensional space by delayed embedding^21^ the point cloud data is distance-measured using the Euclidean method, and the Rips complex shape is constructed using the Vietoris-Rips algorithm and returned to generate the persistence topological features, i.e., the persistence diagram^20^ and the persistence diagram vectorization is represented as a persistence image to more intuitively visualize and analyze the results of persistence topology. The structure of the high-order PI is shown in Fig. 2.

### Generators

The generator in the PIGAT-GAN model utilizes multiple modules, as shown in Fig. 1. The generator includes two layers of GAT, one layer of LSTM, and one layer of Fully Connected Layer (FC). The first layer of GAT is configured with 8 heads, which first receive the node feature matrices and adjacency matrices of the high-order PIs for feature extraction operations, learning several different feature subspaces in parallel. A single-head structure is used in the second GAT layer to integrate features learned at different scales from the individual heads of the first layer, continuously aggregating new node features in this way.

Given $\:N$ node features, the attention coefficient it can be defined as follows:

1 $${\mathrm{e}}_{{{\mathrm{ij}}}} = {\mathrm{a}}\left( {{\mathrm{W}}h_{{\mathrm{i}}} ,{\mathrm{W}}h_{{\mathrm{j}}} } \right)$$

where $\:W$ is the parameter to be learned, $h_{i}$ and $h_{j}$is the node characterization.

Normalizing the attention coefficients using the $\:SoftMax$ function, The final normalized attention coefficient can be defined as follows:

2 $${\mathrm{a}}_{{{\mathrm{ij}}}} = {\mathrm{softmax}}({\mathrm{LeakyReLU}}({\mathrm{e}}_{{{\mathrm{ij}}}} ))$$

The experimental design uses two layers of GAT with K(K = 8) head and single-head attention mechanism strategies, respectively^25–27^. This process can be defined as follows:

3 $$\vec{h}_{i}^{'} = \sigma \left( {\frac{1}{K}\mathop \sum \limits_{{k = 1}}^{K} \mathop \sum \limits_{{j \in N_{j} }} a_{{ij}}^{k} W^{k} \vec{h}_{j} } \right)$$

EEG time series have obvious temporal correlation, in order to effectively capture this temporal property, this study uses LSTM for feature extraction.LSTM is a practical variant of recurrent neural networks^28,29^ which solves the problem of gradient vanishing that occurs in long sequence training of traditional RNN by introducing a gating mechanism^28,30^. The core of LSTM lies in its unique memory cell and three gating mechanisms^31^: (1) forgetting gate: decides what information to discard from the memory cell; (2) input gate: controls the updating of new information; (3) output gate: determines the output of the next time step.In this study, the LSTM network receives the feature sequence extracted by the PI as input, and the network is processed through time-step-by-time-step processing, and finally the implicit state of the last time step is used as the feature representation of the whole sequence.This implicit state contains information about the temporal characteristics of the input sequence, which is subsequently fed into the fully connected layer for classification prediction.The fully connected layer uses a Sigmoid activation function to map the features extracted by LSTM to the [0,1] interval and outputs the final prediction results.

**Fig. 3:**
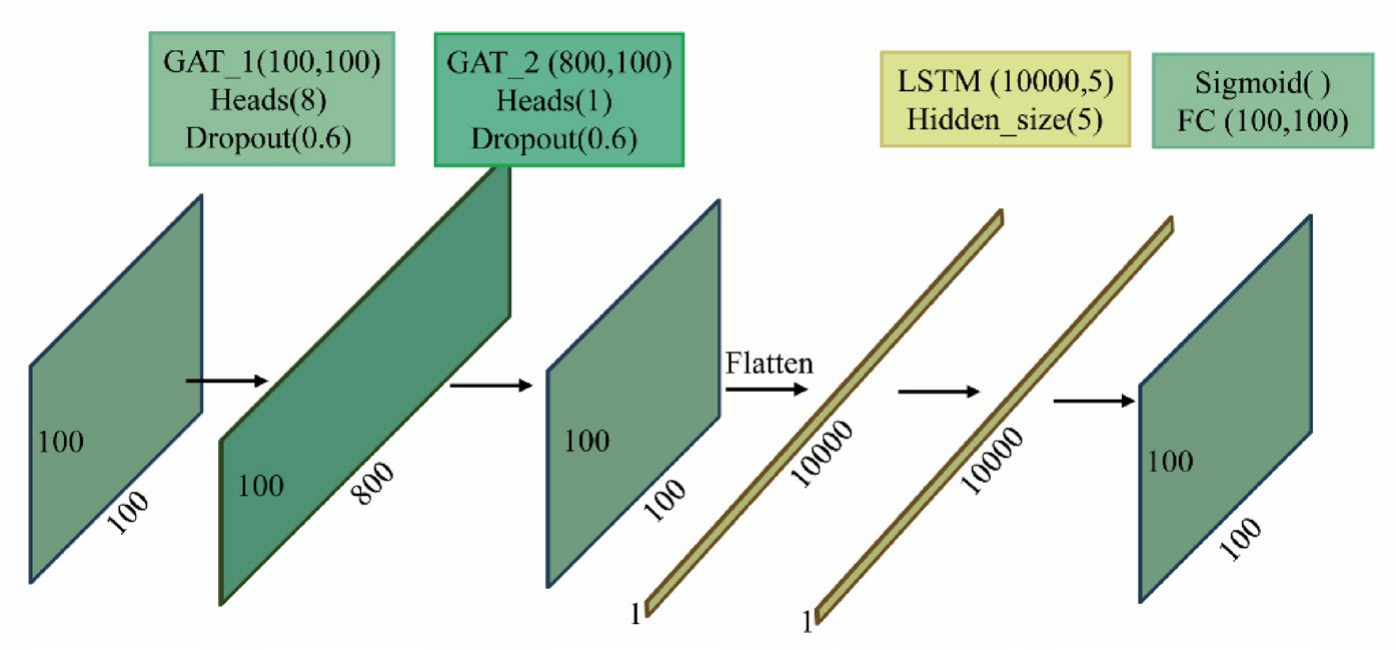
The transformation process of the persistence images through the generator.

The EEG time series preprocessing yields a high-order PI with a shape of 100*100, and after two layers of GAT, the size of the image remains the same, still 100*100. In addition, the random deactivation (Dropout) ratio is set to 0.6 during training. After passing through the GAT layer the image is converted into a shape of 10,000*1 to be fed into the LSTM layer, the dimension of the hidden state vector in the LSTM is set to 5, and after that, the output is reshaped into a shape of 100*100 by passing through the FC layer, Fig. 3 demonstrates the process of transforming the PI through the generator. By looking at this diagram, you can get a clear picture of how the data flows and is processed in the generator.

### Discriminators

In the PIGAT-GAN model, the discriminator evaluates the difference between the predicted generated high-order PI and the actual high-order PI by comparing them and feeding back to the generator, which causes the generator to continuously adjust its parameters to improve the fidelity of the generated PI, which in turn improves the prediction accuracy of the discriminator.

The predicted PIs are processed through two layers of FC in the discriminator, where the PIs are multiplied with the weights as input nodes and then summed, and the predictions are mapped between 0 and 1 by the Sigmoid activation function. The FC layer can be defined as follows:

4 $$\:{Y}_{d}=Sigmoid({{W}_{d}\cdot\:V}_{d}+{b}_{d})$$

where $\:{W}_{d}$ denotes the weights of the discriminator model, $\:{b}_{d}$ denotes the bias, and $\:{V}_{d}$ and $\:{Y}_{d}$ denote the predicted PI results and the true results output by the generator model, respectively.

## Results and analysis

### Data sets and evaluation indicators

The experimental data consisted of 59 channels of EEG recordings in the resting state with eyes closed, provided by Beijing Huilongguan Hospital^32^. The dataset consisted of recordings from 103 patients with schizophrenia and 92 healthy subjects, distributed by gender, age, and years of education. Statistical information on the clinical data of the two groups of subjects is shown in Table 1. Two additional clinical data sets were provided, including PANSS total score, positive score, and negative score.

Data were collected using NeuroScan’s 64-lead EEG acquisition device with impedance kept below 5 kΩ, ground electrodes were AFz, and reference electrodes were physically connected to the right and left mastoids; vertical EEG recordings were made by placing the electrodes above and below the left eye, and horizontal EEG recordings were made by placing the electrodes at the orbital rim of the right eye.

**Table 1:** Demographic and clinical data of the two groups of subjects.

Features	Schizophrenia (*n* = 103)	Healthy subjects (*n* = 92)	Statistical value
Average age	30.55 ± 8.002	30.55 ± 7.287	*F*_1,195_ < 1
Education	14.17 ± 2.494	14.79 ± 2.557	*F*_1,195_ = 3.022, *p* = 0.084
Sex (m/f)	51/52	48/44	χ_1_^2^ = 0.182, *p* = 0.670
PANSS total score	74.31 ± 10.87	–	–
Positive score	20.53 ± 4.72	–	–
Negative score	17.18 ± 5.63	–	–

Raw EEG data were preprocessed using the EEGLAB toolbox. First, after importing the data, positioning the electrodes and performing a re-reference operation. Then, a filtering operation was performed on the EEG signal, applying 1–5 Hz bandpass filtering and 50 Hz trap filtering. Interference from ophthalmic and electromyographic sources was removed using ICA (independent component analysis)^33,34^. Bad leads were inserted and bad segments were eliminated s^32^. The data were then categorized into Delta (1–3 Hz), Theta (4–7 Hz), Alpha (8–12 Hz), Beta (13–30 Hz), and Gamma (31–49 Hz) frequency ranges. The experiment intercepted data from 40 to 200 s, i.e., 160 s and deleted data from other time periods, and used the Giotto.tda toolbox to extract higher-order persistence images for each frequency band, and conducted the experiment using 160 s of EEG data for each subject, divided into 4 s each.

The experiments were conducted to assess the performance of the prediction model using Area Under the Curve (AUC), Mean Average Precision (MAP), and Mean Absolute Error (MAE). MAE and MAP can be defined as follows:

5 $$MAP = \frac{{\sum\nolimits_{{i = 1}}^{K} {AP_{i} } }}{K}$$

6 $$MAE = \frac{{\sum\nolimits_{{i = 1}}^{n} {\left| {y_{i} - \hat{y}_{i} } \right|} }}{n}$$

where $\:{y}_{i}$ is the predicted value of the model, $\:{\widehat{y}}_{i}$ is the actual value, $\:P$ is the model prediction precision, $\:R$ is the recall, and the area under the $\:P-R$ curve is the precision of the model prediction, denoted as $\:AP$.

### Experimental setup

In the experiment, the RMSprop optimizer was used for model training, and all parameters were determined through systematic adaptation. The specific experimental configuration is as follows: During the generator pre-training phase, the learning rate is automatically set to 0.01 through optimization, which can significantly accelerate initial convergence. In the generator-discriminator collaborative training phase, the learning rate is dynamically adjusted to 0.001, and training stability is ensured through real-time gradient balance monitoring. The batch size was determined to be 32 based on memory efficiency analysis, and the classification threshold was set to 0.4. The model uses a binary cross-entropy loss function, which exhibits excellent probability calibration characteristics in binary classification tasks. The dropout rate of the GAT layer is set to 0.5, which has been verified by ablation experiments to reduce the risk of overfitting. The dataset was strictly divided in accordance with EEG research standards^35^ with 80% of the schizophrenia samples used as the training set and the remaining 20% used as the test set. From the 80% training set, 10% was selected as the validation dataset. Data preprocessing was performed in a computing environment with 64 CPU cores and 512 GiB of memory. The pretraining and training processes were implemented using the PyTorch framework in a Python 3.8 environment.

### Predicted results

**Fig. 4:**
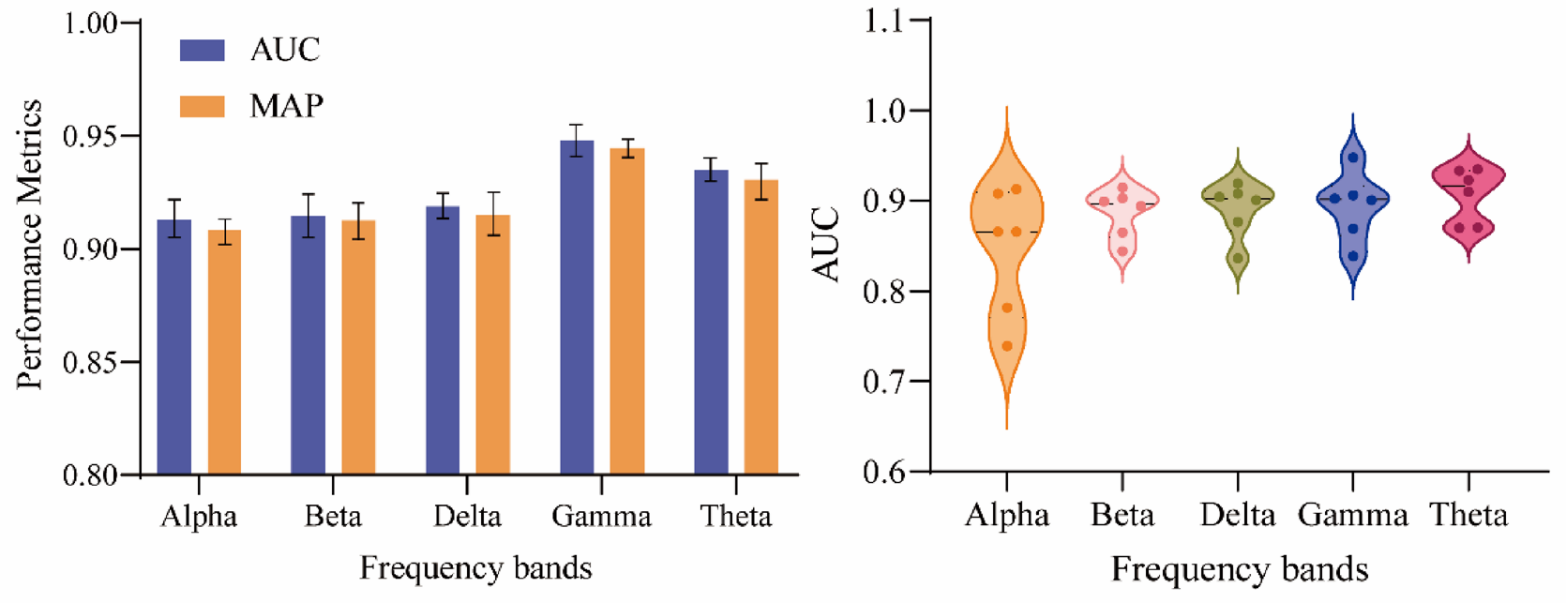
Maximum AUC and MAP values at five frequency bands.

To verify the effectiveness of the PIGAT-GAN model in the processing of schizophrenia EEG signals, the experiments trained the schizophrenia EEG signals on five frequency bands separately, and the prediction results of each band were obtained, Fig. 4 shows the highest values of AUC and MAP in the five frequency bands with different number of samples. Among them, the Gamma band had the best AUC and MAP, reaching 94.7% and 94.4%, and the Theta band showed superior performance at all sample sizes, with an average accuracy of 91.5%, outperforming the other bands.

**Fig. 5:**
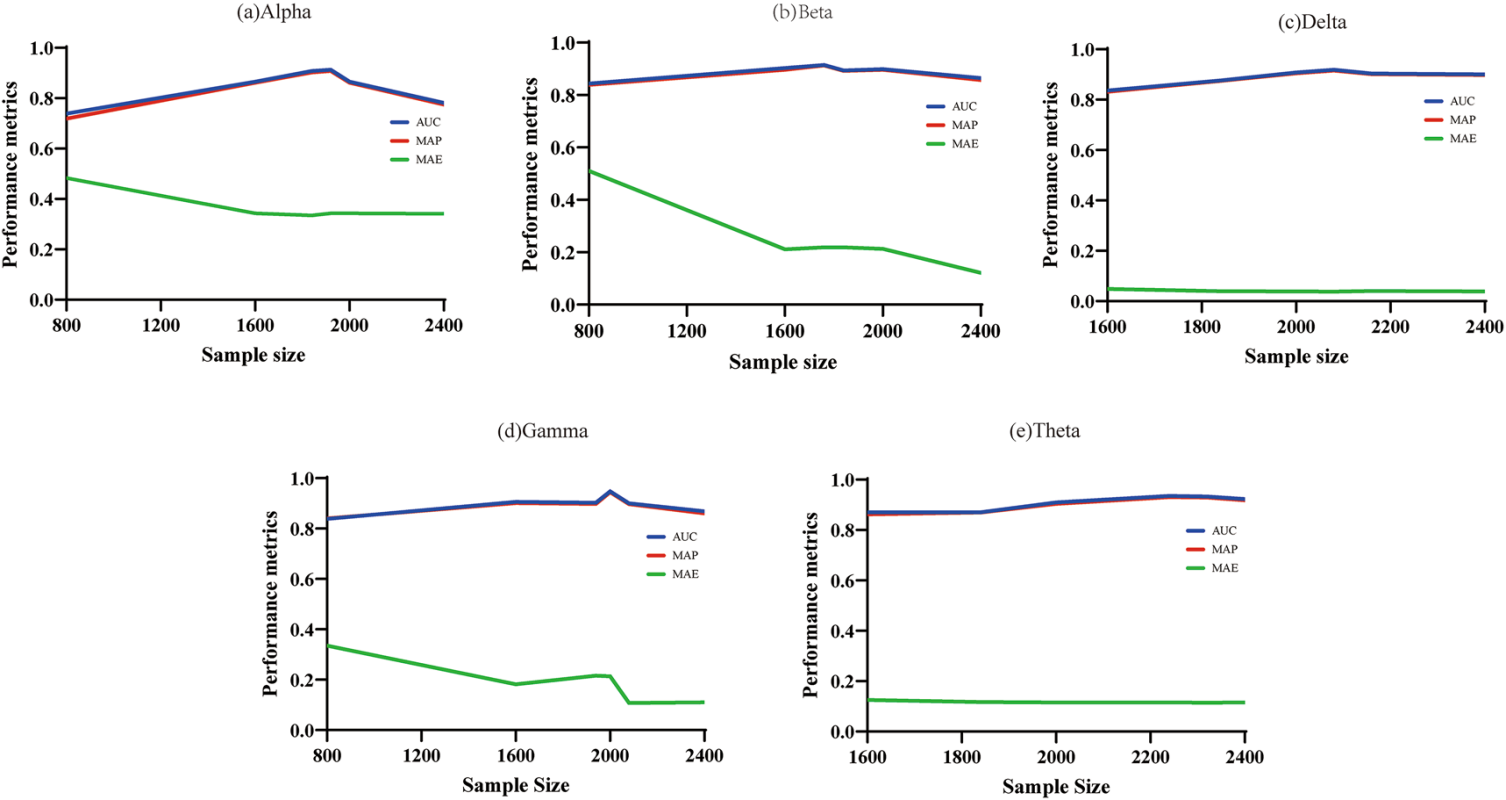
Processes of change in the performance of the five frequency bands.

As can be seen from the data in Exhibit S1, the predictive performance of the model shows a nonlinear change pattern, with differences in AUC, MAP and MAE for different sample sizes. In order to explore the relationship between these differences and the sample size more deeply, we tested different frequency bands with different sample sizes, as shown in Fig. 5, which demonstrates the performance change process of the five frequency bands in detail. The prediction performance exhibits the characteristic of enhancing and then weakening with the increase of sample size. In the initial stage of model training, the discriminative ability of the classifier improves significantly as the amount of available data increases, and both evaluation indexes show a steady increase; however, when the sample size exceeds a specific threshold, the model’s performance begins to gradually decrease. This pattern of change in predictive efficacy with data volume can be interpreted from multiple perspectives. From the data level, systematic errors and random noise may accumulate during the acquisition process, leading to a decrease in signal quality. From a pathological perspective, the experimental group of subjects showed statistically significant differences from the control group in the variability of brain functional network characteristics due to disease factors. With the expansion of the sample size, such pathological difference features may be further amplified, making it difficult for the model to accurately extract discriminative feature representations, which ultimately leads to a decline in generalization performance.

### Modelling losses

**Fig. 6:**
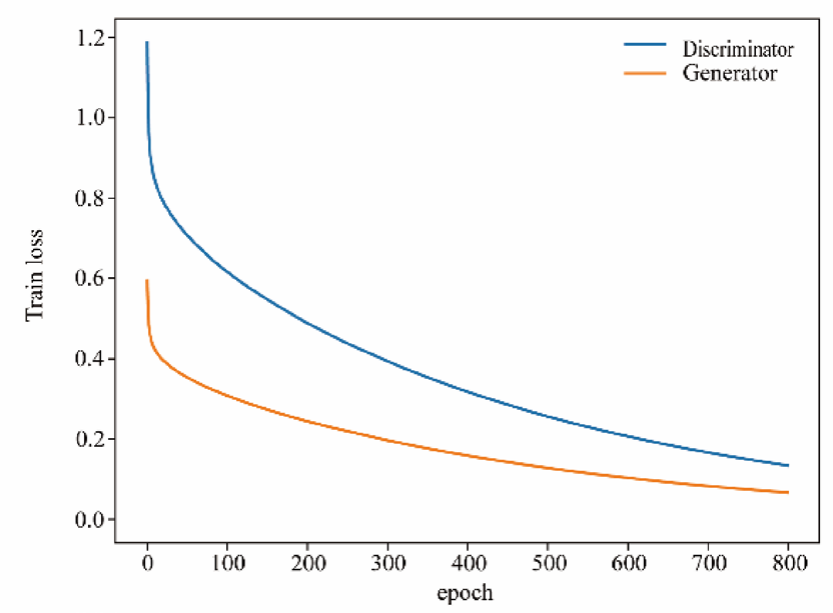
Theta band loss value change.

As the number of training rounds gradually accumulates, observe the process of change in the loss of the generator and discriminator in terms of binary cross-entropy. As shown in Fig. 6, which shows the change curve of the loss value of the Theta band in schizophrenia patients in the first 800 rounds of training, the loss of both the generator and the discriminator showed a significant decreasing trend as the number of training rounds increased.

### Ablation experiments

**Fig. 7:**
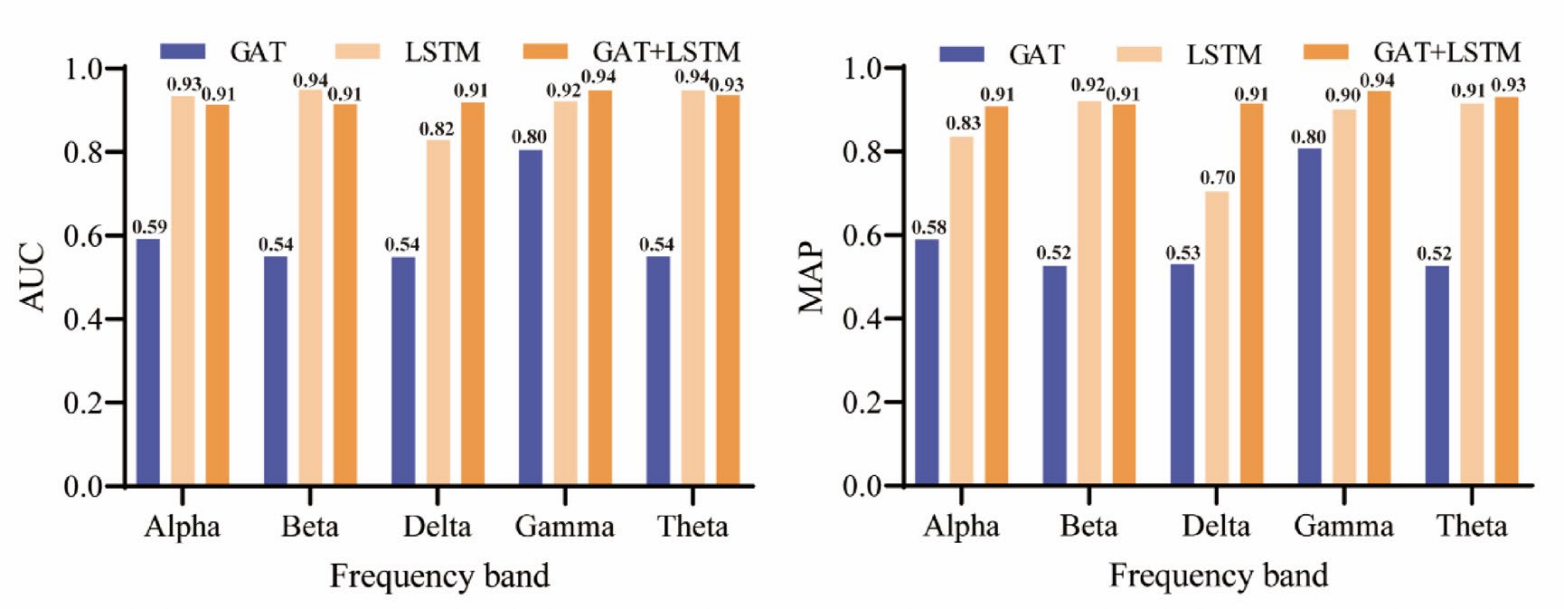
Results of ablation experiment.

The GAT module effectively captures the relationships between nodes through the graph attention mechanism, which is particularly important when dealing with complex spatial dependencies and non-Euclidean data structures; the LSTM module, with its powerful time-series modelling capability, is able to capture the temporal dynamics and long-term dependencies in the data.The combination of the GAT and LSTM modules, through the synergistic effect of space and time, improves the model’s overall performance, enabling more accurate predictions using information in both spatial and temporal dimensions. To verify the importance of GAT and LSTM modules in the model, we conducted ablation experiments to assess their impact on the model performance by removing GAT and LSTM modules from the generator, respectively. The results of the experiments are shown in Fig. 7, where a significant decrease in both AUC and MAP values is observed when only the GAT module is included in the model, which further demonstrates the critical role of the LSTM module in capturing temporal features. Similarly, when only the LSTM module is retained in the model, the AUC and MAP values also decrease, but the decrease is relatively small, which indicates that the GAT module has an irreplaceable role in spatial feature extraction. Taken together, the combination of GAT and LSTM modules significantly improves the performance of the model through the synergistic effect of space and time, verifying the complementarity and necessity of the two in the PIGAT-GAN algorithm.

### Comparative experiments

In order to verify the superiority of the proposed method, experiments were conducted to compare the PIGAT-GAN model with other models, including Support Vector Machine (SVM), GAN, Random Forest, Convolutional Neural Network (CNN) and Recurrent Neural Network (RNN). All models have preprocessed continuous images as inputs, which are generated through topological data analysis conversion of raw EEG signals. The experimental results are shown in Table 2, compared with the other five methods, the PIGAT-GAN model is higher than the other models in both AUC and MAP indicators.

**Table 2:** Comparison of prediction accuracy of different algorithms.

Methods of comparison	Evaluation indicators	Alpha	Beta	Delta	Gamma	Theta
SVM	AUC	81.25	71.87	65.62	87.50	63.06
	MAP	81.00	86.47	58.78	92.50	63.53
GAN	AUC	68.63	68.45	68.40	73.79	73.80
	MAP	65.68	65.43	65.28	69.95	69.78
Random forest	AUC	73.33	75.00	77.14	76.56	85.42
	MAP	89.68	83.33	84.33	88.33	89.72
CNN	AUC	71.88	75.71	51.25	90.00	65.15
	MAP	81.06	58.56	59.08	94.15	84.25
RNN	AUC	84.83	82.55	85.01	92.91	90.62
	MAP	87.50	86.52	86.80	93.50	93.80
PIGAT-GAN	AUC	**91.28**	**91.45**	**91.88**	**94.78**	**93.50**
	MAP	**90.82**	**91.25**	**91.51**	**94.46**	**93.06**

Best evaluation indicators and frequends are indicated in bold.

### Statistical analysis

For statistical analysis, feature vectors of high-order PIs were extracted using convolutional neural networks and used as inputs for regression prediction, and the Adam optimizer was used during the training process, and the results of 500 rounds of training were used as the image quality coefficients^36^ for each sample.

To investigate the significant relationship between the image quality coefficients and the clinical score values of patients with schizophrenia, the image quality coefficients and the clinical score values of each frequency band were statistically analyzed, and as shown in Table 3, there was a significant correlation between the image quality coefficients of the Gamma and Theta frequency bands and the PANSS total score.

**Table 3:** Correlation between image quality coefficients and clinical score values.

Clinical score values	Alpha	Beta	Delta	Gamma	Theta
PANSS positive score	0.0545	0.0599	0.8916	0.6132	0.8638
PANSS negative score	0.3087	**0.0088**	0.5083	0.0682	0.5856
PANSS total score	0.1970	0.5994	0.8040	**0.0166**	**0.0215**

Best evaluation indicators and frequends are indicated in bold.

## Discussion

In this paper, the PIGAT-GAN model is proposed for analyzing and predicting patients with schizophrenia, which extracts the high-order persistent topological features of the EEG time series, and the study divides the EEG signals into five frequency bands, and extracts the high-order PIs using the topological data analysis method, which is combined with the GAN to make the prediction. Prediction experiments are conducted on a schizophrenia dataset and the experimental results show that the Theta band has the best performance with an average accuracy of 91.55% for different samples. In this study, the performance of the model is discussed from three aspects.

First, the experiment analyzes the effect of different sample numbers on the performance of the PIGAT-GAN model under five frequency bands, and we find that there are certain differences in the performance of the model under different sample numbers, and the increase of sample numbers has a positive effect on the improvement of the model’s performance, but when the number of samples exceeds a certain value, the improvement of the model’s performance gradually tends to level off.

Secondly, to improve the validity of the model, this study did ablation experiments to remove the GAT and LSTM modules from the generator, respectively. The results show that the AUC and MAP of the model show a significant decline when only the GAT module is included in the generator, while the AUC and MAP also decrease when only the LSTM module is included in the generator, but the decline is relatively small. Further highlighting the important role of the GAT and LSTM modules in the model, an effective combination of the two can achieve superior predictions across the five frequency bands compared to the use of GAT or LSTM alone.

Finally, the performance of this study using the PIGAT-GAN method is significantly better than the other compared methods since GAT can better capture the complex feature relationships between nodes, leading to a more accurate understanding of the intrinsic structure of the data. Meanwhile, the generator of the PIGAT-GAN model has excellent predictive ability to generate accurate PIs, and it is not difficult to see that this result indirectly validates the effectiveness of the extracted PIs in this study in terms of distinguishing between patients and subjects with schizophrenia, even though the predictive performance of the traditional machine learning methods SVM, Random Forests, and GAN is lower compared to the PIGAT-GAN method. By combining CNN and regression modeling, the image quality coefficients of the patients were determined, and more importantly, it was found that the image quality coefficients in the Gamma and Theta bands were significantly correlated with the PANSS total scores of patients with schizophrenia.

## Conclusion

In this study, we proposed a new prediction method for schizophrenia by extracting high-order PIs and using the PIGAT-GAN model to make predictions and achieved good prediction results. The experimental results demonstrate its effectiveness in capturing complex spatial and temporal features, and the proposed prediction model performs best in the Theta band, with AUC and MAP values of 93.5% and 93.0%, respectively, and an average accuracy of 91.5%. In addition, the image quality coefficients of patients with schizophrenia were significantly correlated with the total PANSS scores in the Gamma and Theta bands, which further validates the potential application of the model in psychiatric assessment.

Some several potential improvements and extensions need to be addressed in future work. First, the current dataset was derived from only one hospital and contained only schizophrenia patients, which may introduce demographic or geographic bias and limit the model’s ability to generalize; future studies need to be extended to multi-center, multi-sample datasets to validate the model’s robustness across different populations and geographic regions. In addition, we plan to explore the applicability of the model in other psychiatric disorders (e.g., depression, bipolar disorder) to validate its generalizability.

## Supplementary Information

Below is the link to the electronic supplementary material.

Supplementary Material 1

## Data Availability

The hospital data used has only the right to use it, and is owned by the hospital. We will communicate with the hospital and share the data subject to all regulations and protocols. Data on the results of this study are available upon reasonable request from the corresponding author.
