# Supplementary material for: Analysis and prediction of schizophrenia patients based on high-order graph attention generative adversarial networks
Source: Sci Rep. 2026 Feb 3;16:4532. doi: 10.1038/s41598-025-15602-8 (PMC12868746; doi:10.1038/s41598-025-15602-8)
Supplement: Supplementary file 1 — Supplementary Material 1 [file 41598_2025_15602_MOESM1_ESM.docx]

In order to verify the effectiveness of the PIGAT-GAN model in the processing of schizophrenia EEG signals, experiments were conducted on five frequency bands of schizophrenia EEG signals, and the prediction results for each frequency band were obtained. Table S1 in the attachment shows the AUC, MAP, and MAE values under different parameters for the five frequency bands. Among them, the AUC and MAP of Gamma frequency band are the best, reaching 94.7% and 94.4% respectively. Theta frequency band performs well in all sample sizes, with an average accuracy of 91.5%, which is better than other frequency bands.

Table S1 Performance analysis of the PIGAT-GAN model for five frequency bands under different samples.

| Alpha | | | | Beta | | | | Delta | | | | Gamma | | | | Theta | | | |
| --- | --- | --- | --- | --- | --- | --- | --- | --- | --- | --- | --- | --- | --- | --- | --- | --- | --- | --- | --- |
| sample | $AUC$ | $MAP$ | $MAE$ | sample | $AUC$ | $MAP$ | $MAE$ | sample | $AUC$ | $MAP$ | $MAE$ | sample | $AUC$ | $MAP$ | $MAE$ | sample | $AUC$ | $MAP$ | $MAE$ |
| 800 | 73.8 | 71.8 | 0.483 | 800 | 84.4 | 83.8 | 0.510 | 1600 | 83.6 | 83.1 | 0.048 | 1600 | 90.6 | 90.1 | 0.182 | 1840 | 87.0 | 86.9 | 0.117 |
| 1600 | 86.5 | 86.0 | 0.342 | 1600 | 90.2 | 89.6 | 0.211 | 2000 | 90.7 | 90.4 | 0.039 | 1840 | 90.2 | 89.7 | 0.216 | 2000 | 90.9 | 90.3 | 0.115 |
| 1840 | 90.7 | 90.2 | 0.334 | 1760 | **91.4** | **91.2** | 0.218 | 2080 | **91.8** | **91.5** | 0.038 | 2000 | **94.7** | **94.4** | 0.213 | 2240 | **93.5** | **93.0** | 0.115 |
| 1920 | **91.2** | **90.8** | 0.342 | 1840 | 89.4 | 89.2 | 0.218 | 2160 | 90.4 | 90.1 | 0.040 | 2080 | 90.0 | 89.6 | 0.107 | 2320 | 93.3 | 92.8 | 0.114 |
| 2000 | 86.5 | 86.1 | 0.343 | 2000 | 89.9 | 89.6 | 0.213 | 2400 | 90.0 | 89.8 | 0.039 | 2400 | 86.9 | 86.0 | 0.110 | 2400 | 92.2 | 91.7 | 0.115 |
